# Microbial diversity and community shifts in a petroleum reservoir under production: effects of water breakthrough and anthropogenic alterations

**DOI:** 10.3389/fmicb.2026.1741638

**Published:** 2026-03-19

**Authors:** Armando Alibrandi, Julia Plewka, Rolando di Primio, Alexander Bartholomäus, Aurèle Vuillemin, Alexander J. Probst, Jens Kallmeyer

**Affiliations:** 1 Section Geomicrobiology, GFZ Helmholtz Centre for Geosciences, Potsdam, Germany; 2 Environmental Metagenomics, Research Center One Health Ruhr of the University Alliance, Essen, Germany; 3 Aker BP, Lysaker, Norway; 4 Faculty of Biology, University of Duisburg-Essen, Duisburg, Germany

**Keywords:** metagenomic profiling, microbial communitydynamics, oil reservoir microbiology, oil reservoir monitoring, reservoir microbiome dynamics, water breakthrough

## Abstract

Subsurface petroleum reservoirs host indigenous microorganisms that survive extreme conditions and long-term isolation. Microbial activity in these environments can contribute to adverse effects such as oil biodegradation and reservoir souring. Unlike the broader deep biosphere, oil reservoirs are frequently subjected to anthropogenic disturbances, particularly during production, when processes like water injection introduce external microbes and electron acceptors. In this study, we investigated microbial diversity, community structure, and the impact of water breakthrough using 16S rRNA gene and metagenomic sequencing of produced fluids, production water, and injection water samples from the Edvard Grieg oil reservoir offshore Norway. We found clear regional heterogeneity in community composition, characterized by overall low diversity, dominated by thermophilic, anaerobic, and halotolerant taxa. The southern region (wells A13, A17, A18, and A19) exhibited lower diversity, while the microbial community composition of well A07 showed a distinct signature. The prevailing genera included the strictly anaerobic bacterium *Thermoanaerobacter* and the hyperthermophilic archaeon *Thermococcus*. Water breakthrough triggered shifts in community structure, not because of widespread replacement by injected microbes, but due to the increase in sulfate-reducing bacteria. Comparison between sequence data from production fluids and water samples allowed the identification of microbial signatures that can act as cost-effective tools for monitoring oil reservoir processes and integrity.

## 1 Introduction

Microbial communities are powerful agents of environmental transformation, capable of altering their surroundings through metabolic activity (Gupta et al., 2017). One of the most economically disruptive microbial processes occurring in oil reservoirs is oil souring, i.e., the production of toxic and corrosive hydrogen sulfide (H_2_S) by microbial sulfate reduction, leading to a diminishing oil value (Magot, 2005; Medina-Bellver et al., 2005; Gieg et al., 2011). In addition to souring, microbial activity degrades the composition and properties of crude oil (Head et al., 2003). Biodegradation increases oil density. In the oil industry, density is commonly expressed as API gravity (American Petroleum Institute), a dimensionless parameter calculated from density and used as a key indicator of oil quality; lower API values indicate higher density and lower-quality oils. A plethora of studies have examined the microbial composition of oil reservoirs (Takahata et al., 2000; Ren et al., 2011; Tang et al., 2012; Lin et al., 2014; Cai et al., 2015; Vigneron et al., 2017), yet most of them focused on samples from flooded reservoirs in which water breakthrough had occurred or was presumed to have happened. In the oil industry, flooding is the process where fluid injections, for offshore reservoirs, mostly seawater as well as formation water, are injected into the reservoir to maintain pressure during the extraction process and increase recovery rates (Clemens et al., 2017). Formation water is naturally present in all oil reservoirs and is extracted alongside crude oil and separated from the oil onboard the oil production platform. Water breakthrough occurs once the injected water reaches the production well, meaning that the recovered oil starts to mingle with the injected fluids. Under such circumstances, microbial community analyses may reflect taxa introduced by water injections rather than indigenous reservoir populations. However, even in non-water flooded reservoirs, contamination from drilling, well operations, or faulty pipes potentially introduces foreign microorganisms, raising the question of whether the microorganisms driving biodegradation and souring are truly native to oil reservoirs or introduced through drilling or well operations (Magot et al., 2000; Youssef et al., 2009; Wentzel et al., 2013).

Water injection, mostly degassed seawater for offshore reservoirs, can transport non-native microorganisms into the reservoirs, and even in small amounts, this can drastically alter microbial communities (Li et al., 2017; Vigneron et al., 2017) given the significantly higher microbial abundance of seawater (10^6^ cells × mL^–1^) compared to typical in-reservoir densities of around 10^2^–10^4^cells × mL^–1^(Magot, 2005). Additionally, water injection into oil reservoirs can lead to marked reductions in *in situ* temperature. In the oil fields Vigneron et al. (2017) studied, temperatures decreased from ∼80°C to as low as 42°C after seawater injection. This shift may create environmental conditions more permissive for microbial activity and proliferation.

Furthermore, seawater used for the injections contains sulfate and phosphate that can foster the growth of sulfate-reducing bacteria (SRB), with subsequent production of H_2_S (Vance and Thrasher, 2005). To mitigate this phenomenon (i.e., souring), nitrates are added to the injection to stimulate the growth of nitrate-reducing microbes that outcompete the sulfate reducers (Larsen et al., 2004; Hubert, 2010).

Beyond their detrimental impact on the value of the oil, microbial communities also hold promise as biosignatures for understanding reservoir dynamics. Recent studies demonstrate that microbial community composition can serve as a tracer of oil migration pathways and fluid provenance, often providing greater precision than traditional geochemical analyses (Zhang et al., 2020, 2021). As sequencing technologies become cheaper, microbial profiling is emerging as a practical tool for oil field monitoring (Wetterstrand, 2019).

To date, the upper-temperature limit for microbial life is documented at 122°C, as demonstrated for *Methanopyrus kandleri* (Takai et al., 2008). However, in oil reservoirs, biodegradation ceases above ∼80°C, as these processes often rely on microbial consortia where disruptions in metabolic linkages can halt the entire process (Wilhelms et al., 2001; Magot, 2005; Head et al., 2014). The exact cause of this temperature threshold remains uncertain, though hypotheses suggest that energy demands for cellular repair in nutrient-limited reservoir environments become prohibitive (Larter et al., 2003). Recently, a study reported that, as microbial cells approach the upper-temperature limit of life in deep, hot subsurface sediments, cellular metabolic rates increase again to counter the thermal degradation of biomolecules (Beulig et al., 2022). Moreover, hydrocarbons impose solvent stress on cell membranes, a challenge that intensifies with rising reservoir temperatures (Pannekens et al., 2019). Combined with high salinity and the abundance of heavy metals, these extreme conditions select for specialized poly-extremophiles (Youssef et al., 2009), primarily from anaerobic and thermophilic clades of Firmicutes (Bacillota), Euryarchaeota, and Thermotogota (Table 1; Wentzel et al., 2013). While some members of these groups, such as *Thermococcus*, can thrive at temperatures exceeding 80°C (Zhao et al., 2015), the combination of multiple stressors in oil reservoirs appears to prevent microbial communities from actively degrading hydrocarbons under such conditions.

**TABLE 1:** Phylum and genera considered to be indigenous to hot oil reservoirs (Wentzel et al., 2013; Head, 2017; Vigneron et al., 2017).

Phylum	Genera
Thermotogota	*Petrotoga, Kosmotoga, Thermotoga, Geotoga, Oceanotoga, Thermosipho*
Firmicutes (Bacillota)	*Thermoanaerobacter, Geobacillus, Bacillus, Desulfotomaculum, Caldanaerobacter, Mahella, Caminicella*
Deinoccocota	*Thermus*
Synergistetes (Synergistota)	*Thermovirga, Anaerobaculum*
Deferribacteres (Deferribacterota)	*Deferribacter*
Euryarchaeota (Methanobacteriota)	*Methanoculleus, Methermicoccus, Methanothermobacter, Methanococcus, Archaeoglobus, Thermococcus*

New(er) nomenclature in parentheses.

Oil reservoir temperatures, dictated by sedimentary depth and geothermal gradients, range from ∼20 to 150°C (Peters et al., 2007). With increasing burial depth, temperatures typically rise at about 3°C per 100 m (Magot, 2005), and in deeper formations, they may exceed 130–150°C, with hydrocarbon accumulations predicted at depths of up to 13 km (Pang et al., 2022). Here, we define “hot reservoirs” as those conditions approaching the 80°C limit for biodegradation (Connan, 1984) rather than the absolute upper temperature limits of microbial life.

Given the pivotal role that microbial communities play in biogeochemical processes within petroleum reservoirs, comprehending their responses to anthropogenic interventions, such as water injection, is paramount in monitoring well evolution during production. Our study aims to elucidate the spatial distribution of microbial communities in Edvard Grieg, a high-temperature (∼78°C) oil reservoir located in the North Sea, and assess the effects of water breakthrough on the structure of the indigenous microbial community in the reservoir.

## 2 Materials and methods

### 2.1 Study site and well sampling for oil and water

Produced fluids (PF), production water (PW), produced oil (PO), and injection water (IW) samples were collected from the Edvard Grieg oil field, located on the Norwegian continental shelf, about 200 km west of Stavanger, Norway. For clarity, we refer to PF as the mixture of crude oil, suspended solids such as sand, and formation water (the saline water originally present in the reservoir). When water breakthrough occurs, PF also contain injection water (seawater and/or separated formation water used to maintain reservoir pressure). We refer to the aqueous phase separated from PF as production water (PW) and the separated hydrocarbon phase as produced oil (PO).

**FIGURE 1:**
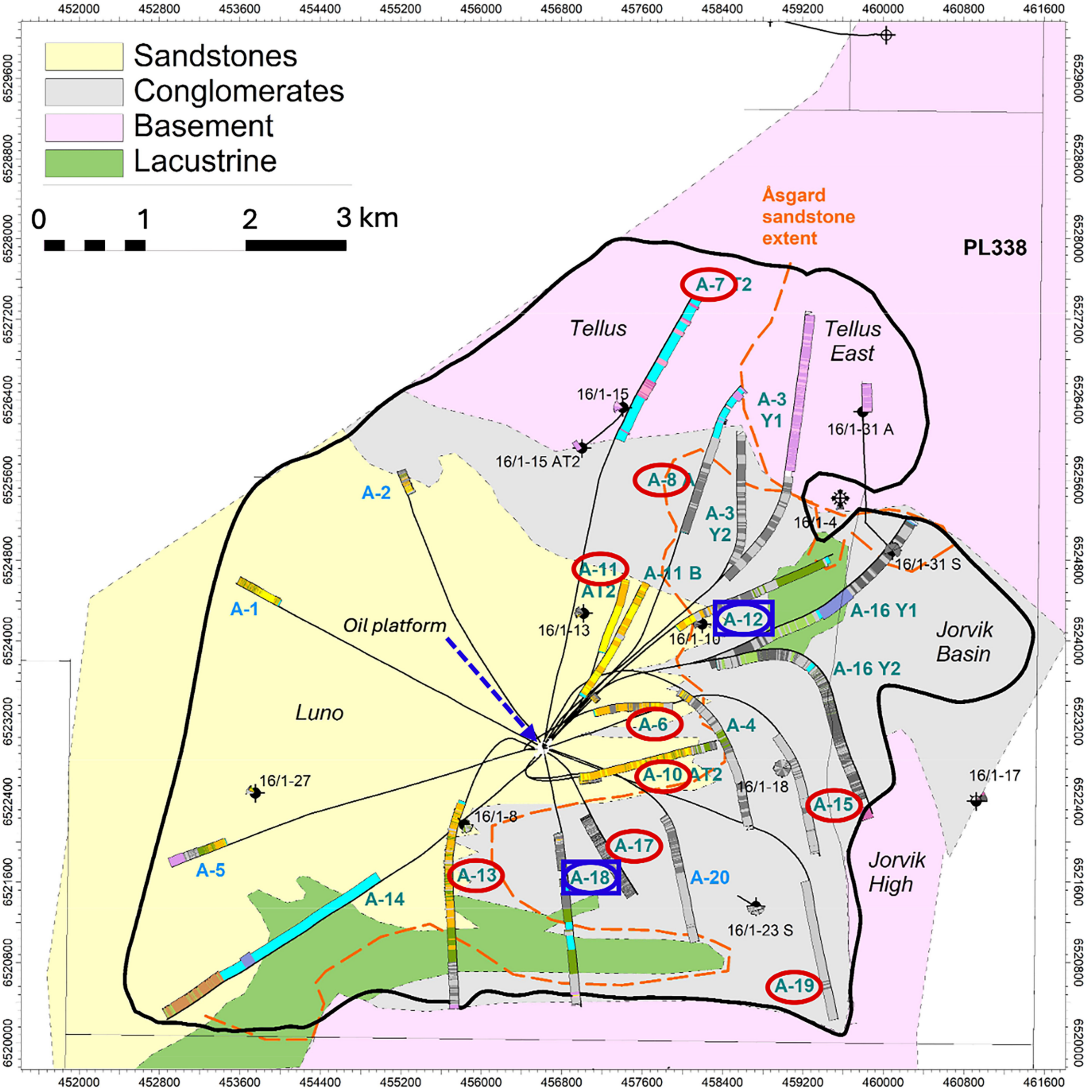
Map of the Edvard Grieg reservoir. The “A” marks denote sampled well numbers. Blue squares represent sites sampled both before and after the water breakthrough, while red circles indicate sites sampled only before the water breakthrough. The black dot at the center marks the location of the oil platform.

The reservoir is located at an average depth of 1,900 m below the seafloor (mbsf), and *in-situ* temperatures range between 76 and 78°C. It is geologically subdivided into three segments, Luno, Jorvik, and Tellus, which are composed of sandstones, conglomerates, and, in part, also weathered granitic basement rocks. Lacustrine shales are non-reservoir sediments in the field (Figure 1). Edvard Grieg was discovered in 2007 and has been in production since 2015.

Produced fluids (PF) were collected from 11 wells (A6, A7, A8, A10, A11, A12, A13, A15, A17, A18, A19). Each of these wells is directly connected to the Edvard Grieg platform (Figure 1). On the day of sampling, the PF from the different wells had a water cut (i.e., fraction of water suspended in the oil) ranging from 1 to 36% (Supplementary Table S3) and API gravity values of 32–34. The high API values of Edvard Grieg correspond to a low density, indicative of a low level of biodegradation.

During the first sampling campaign, water injections had been ongoing for 1–2 months, but a water breakthrough had still not occurred. Sixteen months later, 1 month after the water breakthrough, a second round of sampling was conducted. Due to operational and logistical constraints, the second campaign was limited to produced fluids only from A12 and A18, which were the only wells made available for re-sampling by the operator. In addition, PO, PW, and IW samples were collected during this campaign.

Injection water was a mixture of oxygen-depleted seawater and separated formation water, supplemented with deposition inhibitors and injected at 40–50°C. Samples were collected at the injection wellhead from a dedicated point on the injection line. The sampling line was flushed for 5 min before sampling. To minimize biofilm formation on the line, the line is regularly treated with biocide every 3 days.

Samples of PW, PF, and PO were collected via standard 8-L miniseparators that are specifically designed for representative sampling of the wellstream. These miniseparators are not operated continuously but are connected directly to the wellstream only when sampling is required, with no intermediate processing equipment between the wellhead and the sampling point. Before sampling, the miniseparator was thoroughly flushed with the wellstream fluid for approximately 2 min at an inlet pressure of 20 bar, ensuring rapid fluid turnover and minimizing carryover. The sampling cylinder was first drained and refilled before the actual sample was taken. Such procedures ensured that samples were representative and minimally influenced by the sampling equipment.

All samples were collected in sterile glass bottles with polytetrafluoroethylene (PTFE) sealed caps and were filled without headspace to maintain anoxic conditions. They were kept at room temperature during transport and, upon arrival, were flushed with N_2_/ H_2_ (99:1) to remove any residual oxygen and stored at 4°C at the home lab until analysis 1–2 weeks later.

### 2.2 DNA extraction

25 mL of PF and PO samples were aliquoted into 50 mL centrifuge tubes inside an anoxic glovebox to prevent oxygen exposure of the remaining samples. DNA extraction followed a modified isooctane method (Alibrandi et al., 2023): samples were mixed with an equal volume of isooctane (2,2,4-trimethylpentane) in a fume hood to minimize exposure to volatile hydrocarbons and solvents, then centrifuged at 5,000 × g. The supernatant was discarded, and the pellet was transferred to bead tubes of the DNEasy PowerSoil Pro kit (Qiagen, Hilden, Germany; Cat. No. 47014) inside a laminar flow cabinet to prevent aerial contamination. To increase DNA yields, 40 μL of 10% sodium dodecyl sulfate (SDS) was added, followed by incubation at 65°C for 10 min before proceeding with the manufacturer’s protocol. Negative controls consisted of the addition of 1 mL of isooctane and 40μL of SDS to the bead tubes, followed by DNA extraction.

Unlike PF and PO samples, PW and IW samples required no cell/particulate separation from the oil matrix, and, therefore, no isooctane method was required. Samples of PW and IW were filtered through Sterivex units (0.22 μm; Merck KGaA, Darmstadt, Germany), filter casings were opened, and the membranes were transferred directly into bead tubes. The same pre-treatment with 40 μL of 10% SDS and incubation at 65°C for 10 min was applied prior to extraction with the PowerSoil Pro kit. Negative controls consisted of DNA extractions from unused Sterivex filters. DNA concentrations in all extracts were quantified using the dsDNA High Sensitivity Assay kit on a Qubit 2.0 fluorometer (Invitrogen, Carlsbad, United States).

### 2.3 Amplification of 16S rRNA genes and amplicon sequencing

All samples were processed in duplicates. Bacterial and archaeal 16S rRNA gene fragments (V4 hypervariable region) were PCR amplified with the universal barcoded primer pair 515F (5’-GTG TGY CAG CMG CCG CGG TAA-3’) and 806R (5’-CCG GAC TAC NVG GGT WTC TAA T-3’). The final volume of each PCR reaction was 50 μL and contained 2 μL DNA template, 0.5 μL Taq DNA polymerase (5U/μL), 2 μL dNTP mix (5 mM), 2 μL MgCl*2* (25 mM), 5 μL 10 × polymerase buffer C, 0.5 μL BSA (20 mg/mL), 2.5 μL of each primer (10 μM), and 33 μL PCR-grade water. All reagents used for PCR were obtained from EurX (Gdańsk, Poland). PCR amplification was run at 95°C for 5 min of initial denaturation, followed by 32 cycles of 30 s at 95°C (melting), 30 s at 56°C (annealing), and 1 min at 72°C (elongation), with a final elongation of 7 min at 72°C. PCR products were cleaned using AMPure magnetic beads (Beckman Coulter, Brea, United States), and barcoded samples were normalized to 20 ng of DNA and pooled. Amplicon sequencing was performed on an Illumina MiSeq platform using 2 × 300 base pair (bp) reads at Eurofins Genomics (Ebersberg, Germany).

### 2.4 16 S rRNA sequence data processing and statistical analysis

Read demultiplexing was performed using Cutadapt (v. 3.5) (Martin, 2011) with the following parameters: -e 0.2 -q 15,15 -m 150 –discard-untrimmed. The amplicon sequence variants (ASVs) were generated using trimmed reads and the DADA2 package (v. 1.20) (Callahan et al., 2016) with R v. 4.1, applying the pooled approach with the following parameters: truncLen = c(220,180), maxN = 0, rm.phix = TRUE, minLen = 160. Taxonomic assignment was done using DADA2 against the SILVA 16S rRNA SSU database release 138 (Quast et al., 2012). ASVs representing chloroplasts, mitochondria, and singletons were removed. The partial 16S rRNA gene sequences were aligned using SINA Online v.1.2.11 (Pruesse et al., 2007) and inserted into a Maximum Likelihood RAxML phylogenetic tree on ARB (Ludwig et al., 2004). ASVs attributed to known bacterial extremophiles and SRB (92 ASVs) and archaea (99 ASVs) were selected and plotted into two separate phylogenetic trees, using the maximum parsimony algorithm with the bacterial and archaeal filters, and selecting the best tree among 100 replicates.

Statistical analyses of alpha and beta diversity were conducted using the Vegan community ecology package in R v. 4.3.1 and the software PAST (v. 4.14; Hammer et al., 2001). The dataset was rarefied to 2,000 reads and included 3,402 out of the total 3,406 ASVs. Downstream statistical analyses were performed with the same rarefied dataset. For a general assessment of the microbial composition, we applied two key metrics: the relative read abundance of ASVs and alpha diversity, including the observed richness and Shannon index. To examine regional differences within the reservoir, we looked at the beta diversity of the microbial composition and conducted a Principal Coordinates Analysis (PCoA) with the Bray-Curtis similarity index, as well as a non-parametric Permutational Multivariate Analysis of Variance (PERMANOVA). The PCoA enabled visualization of the dissimilarity between microbial communities, providing insights into clustering patterns and trends across different regions. PERMANOVA was used to determine the statistical significance of the observed differences.

To investigate microbial community dynamics within the reservoir before and after injection, we combined PCoA and PERMANOVA approaches, as described above, with the Indicator Value (IndVal) Analysis to assess overall shifts in microbial composition and identify which specific microbial taxa can be significantly associated with the pre-water breakthrough PF, post-water breakthrough PF, PW, PO, and IW samples. This method considers both relative species abundance and occurrence frequency to determine the specificity and fidelity of a given group. For the calculation, we only used ASVs that had a cumulative count of more than 100, resulting in 29 selected ASVs.

To assess whether the water cut of the oils influenced the microbial composition, we ran a PERMANOVA test to see if the microbial composition varies with the water cut and ran a Mantel test to measure the correlation between the microbial structure and the water cut. Additionally, we performed non-metric multidimensional scaling (NMDS) based on Bray-Curtis dissimilarity to visualize patterns in microbial community composition across samples. The stress value was reported to indicate the goodness of fit of the ordination ( < 0.2 indicates a good fit). To test whether specific ASVs are influenced by the water cut, a generalized additive model (GAMs) was applied (v. 1.9-3) (Wood, 2017) using the R package mgcv. Significance was obtained by *p*-value < 0.05 corrected by the FDR method.

### 2.5 Metagenomic sequencing, *de novo* assembly, and gene annotation

The same DNA extracts used for 16S rRNA gene sequencing were sent to CeGaT GmbH (Tübingen, Germany) for metagenomic sequencing. Libraries were prepared using the Nextera XT DNA Library Preparation kit (Illumina), and sequencing was performed on a NovaSeq 6000 Illumina platform, aiming for 50 million read pairs (2 × 150 bps). Functional annotations of predicted Open Reading Frames (ORFs) were extracted using prodigal (Hyatt et al., 2010) and functionally assigned using the software DIAMOND protein aligner (v. 0.9.24; Buchfink et al., 2015). Proteins were annotated with eggNOG v. 2.1.12 and eggNOG DB (v. 5.0.2; Cantalapiedra et al., 2021; Huerta-Cepas et al., 2019). We performed a quantitative functional annotation, focusing on open reading frames (ORFs) encoding proteins predicted to function in sulfate, sulphite, and polysulphide reduction; nitrate and nitrite reduction; hydrocarbon degradation; methanogenesis; heat shock response; salt stress; biofilm formation; and microaerophilic respiration (Supplementary Tables S2). We ran a PERMANOVA analysis to assess whether the differences between the ORFs of interest associated with the sample groups were significant.

### 2.6 Principal coordinate analysis based on gene sequence coverage

BBDuk (v. 37.09; Bushnell, 2014) removed Illumina artifacts and adapters from the shotgun metagenomic raw reads. We trimmed the reads based on the quality scores with Sickle (v. 1.33; Joshi and Fass, 2011) and deduplicated them with BBMap (v. 37.09; Bushnell, 2014). We assembled the quality-controlled reads with metaSPAdes (v.3.15.5; Nurk et al., 2017). Afterward, all scaffolds were filtered with at least 1,000 base pairs using pullseq (v. 1.0.2; (Bushnell, 2014), which were then used for gene prediction via prodigal (Hyatt et al., 2010). The nucleotide sequences of predicted genes from all metagenomes were clustered with MMSeqs2 (v. 15.6f452) (Steinegger and Söding, 2017) in cluster-mode 2, coverage-mode 1, minimum breadth of 95% and minimum sequence identity of 95%. Quality-controlled reads were mapped against the representative sequences of the resulting clusters with bowtie2 (v. 2.3.5.1) (Langmead and Salzberg, 2012) in sensitive mode. Sequences were counted as present in samples if the minimum coverage breadth was > 95% and coverage > 5. The coverage values were normalized based on base pair counts of the sequenced forward reads. Only gene sequence clusters present in more than one metagenomic sample were used for the PCoA calculation to avoid the influence of undersampling. The PCoA was visualized with the R (R Core Team et al., 2020; RStudio Team, 2020) package ggplot (Wickham and Wickham, 2016). The upSet plot was created with the package ComplexUpset (Krassowski et al., 2022).

### 2.7 Prokaryotic community composition based on extended rpS3 gene sequences

The ribosomal protein S3 (rpS3) marker gene was used to estimate the prokaryotic community composition (Sharon et al., 2015). Marker genes were identified with species-specific Hidden Markov Models (HMMs) and by comparing the amino acid sequences of predicted genes with diamond blastp (Buchfink et al., 2015) against the UniRef100 database (downloaded on 29.10.2023) (The UniProt Consortium, 2019) with an *e*-value cut-off of 1e-5. The rpS3 gene nucleotide sequences with 1,000 bps flanking regions were extracted for all samples and clustered with MMSeqs2 (v. 15.6f452) (Steinegger and Söding, 2017) in cluster-mode 2, coverage-mode 1, minimum breadth of 95%, and minimum sequence identity of 95%. RpS3 gene sequences were taxonomically annotated by comparing them with rpS3 sequences extracted from the GTDB (GTDB v. 220) (Parks et al., 2022) with usearch -ublast (v. 10.0.240_i86linux64) (Edgar, 2010). Sequences that could not be annotated via this approach, but that were binned, were assigned the MAG taxonomy (by finding the bin that carried the scaffold with the rpS3 gene sequence; for MAG construction, see below). Quality-controlled reads of all samples were mapped against the representatives with bowtie2 (v. 2.3.5.1) (Langmead and Salzberg, 2012) in sensitive mode. Reads mapping with more than 10% mismatches were excluded. The mean coverage depth of extended rpS3 sequences was calculated for all sequences with a coverage breadth > 95%. Coverage was normalized by the base pair count of the forward reads. The data was visualized in R (R Core Team et al., 2020) with the RStudio interface (RStudio Team, 2020). DNA samples were sent to CeGaT GmbH (Tübingen, Germany) for metagenomic sequencing. Libraries were prepared using the Nextera XT DNA Library Preparation kit (Illumina), and sequencing was performed on a NovaSeq 6000 Illumina platform at CeGaT, aiming for 50 million read pairs (2 × 150 bps). Due to the extremely low biomass across all PF samples, DNA yields were very low for metagenomic library preparation. Only six samples marginally fulfilled the minimum DNA input requirements and could therefore be successfully processed for metagenomic sequencing: well A18 before injection, wells A12 and A18 after injection, production water, and injection water. Biological replication was limited by biomass constraints; only the A18 post-injection sample was sequenced in biological duplicate, whereas all other samples were sequenced once. These samples were not selected based on biological criteria but solely on technical feasibility. Consequently, the metagenomic dataset represents a subset of technically viable samples rather than wells with distinctly higher biomass or divergent community types. All remaining samples yielded insufficient DNA for metagenomic sequencing but were successfully analyzed using 16S rRNA gene amplicon sequencing.

### 2.8 Reconstruction of metagenome-assembled genomes

Scaffolds with a minimum length of 1,000 bp were binned into MAGs using ABAWACA (v. 1.0.0) (Brown et al., 2015) and MaxBin2 (v. 2.2.7) (Wu et al., 2016) with default parameters. The bins were aggregated with DASTool (v. 1.1.6) (Sieber et al., 2018) and the resulting selection was manually curated in uBin (v. 0.9.14) (Bornemann et al., 2020). Completeness and contamination of curated MAGs were calculated with CheckM2 (v. 1.0.1) (Chklovski et al., 2023) and used GTDB-tk (v. 2.4.0) (Chaumeil et al., 2022) with the Genome Taxonomy Database (v. 220) (Parks et al., 2022) to assign taxonomy. The taxonomic annotation was used to build a *de novo* tree of the MAGs with GTDB-tk (Chaumeil et al., 2022) *de_novo*_wf workflow, converted into an iTOLs usable format with GTDB-tk’s convert_to_itol workflow, and visualized with iTOLs (v. 6) (Letunic and Bork, 2024).

ANI analysis was performed using FastANI (Jain et al., 2018) to compute the pairwise average nucleotide identity among the Thermoanaerobacter genomes. This tool identifies orthologous genomic fragments via bidirectional mappings and calculates the percentage of nucleotide similarity. Following established criteria, genomes with an ANI > 95% were considered to belong to the same species. Despite the fragmented nature of some assemblies, which may affect fragment ordering, the ANI values provide a robust measure of overall genomic similarity.

### 2.9 Prediction of putative viral scaffolds and strain clustering

Putative viral scaffolds in the metagenomes were identified using three different tools: VIBRANT (v. 1.2.1) (Kieft et al., 2020); VirSorter2 (v. 2.2.4) (Guo et al., 2021) in sensitive mode; and DeepVirFinder (v.1.0) (Ren et al., 2020) with a threshold of 0.7. Hosts’ contaminations were removed, and completeness was calculated using CheckV (v. 1.0.1) (Nayfach et al., 2021). Only scaffolds, predicted as viral by all three tools, with predicted completeness ≥ 25% and no warnings by CheckV v. 1.0.1, were used for further analyses. In total, 31 putative viral scaffolds were identified (Supplementary Table S10). Those were mapped with Bowtie2 (v. 2.4.1) (Langmead and Salzberg, 2012) and samtools (v. 1.13) (Li et al., 2009) against all quality-controlled raw reads. We used the mappings to calculate and compare single-nucleotide polymorphism (SNPs) and average nucleotide identity (ANI) with InStrain (v. 1.8.1) (Olm et al., 2021) between the viral strains. Viral taxonomy was predicted with genomad (v. 1.11.2) and genomad database (v. 1.9) (Camargo et al., 2024). Viral host pairings were predicted using iPHoP (v. 1.4.2) (Roux et al., 2023) after adding the MAGs curated from the metagenomes to the database.

## 3 Results

### 3.1 Microbial diversity and spatial variation

**FIGURE 2:**
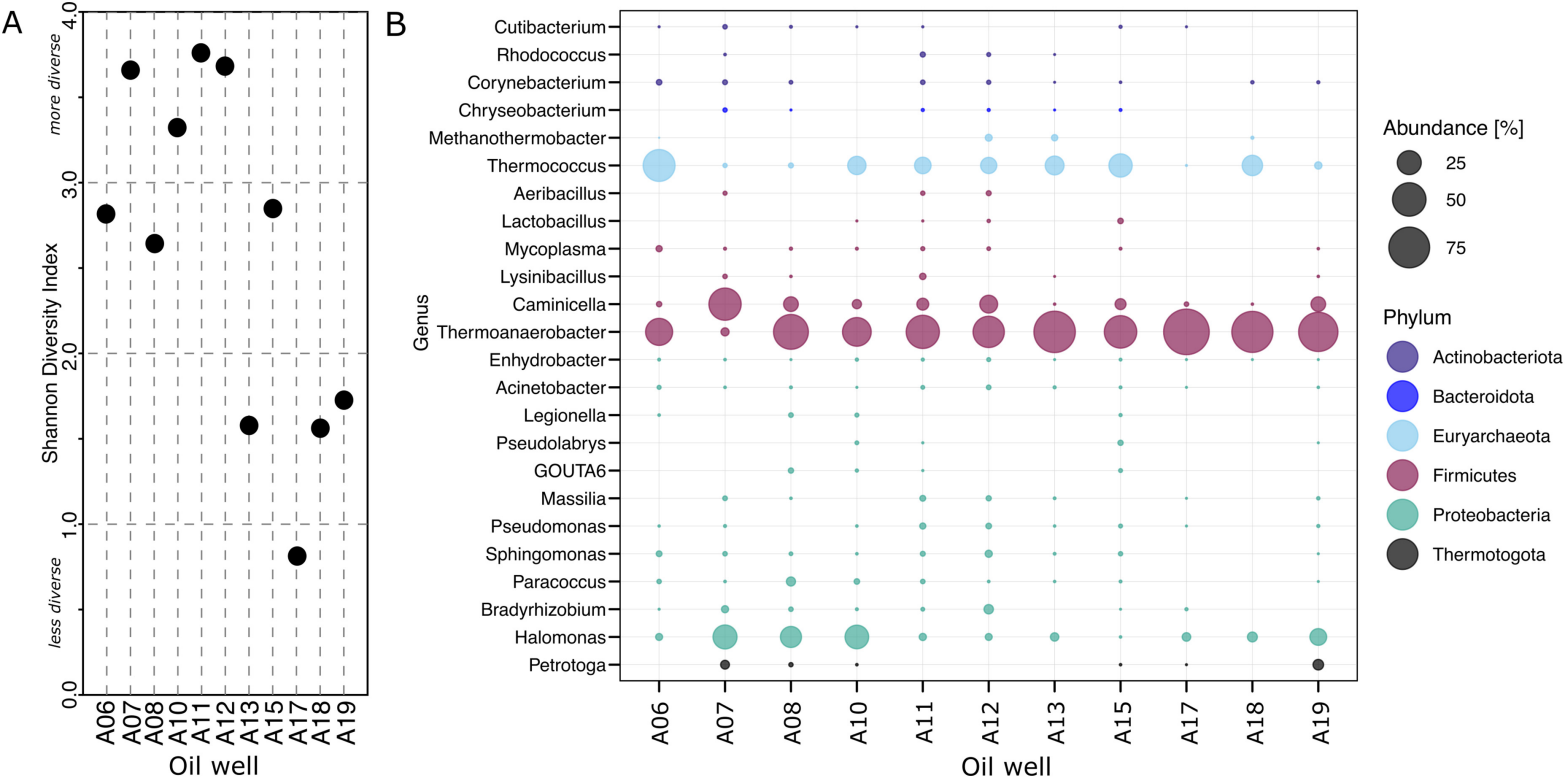
Abundance and diversity obtained by the produced fluids of Edvard Grieg Reservoir. **(A)** Visualization of the Shannon index. **(B)** The bubble plot shows 94% of the most abundant taxa. Each bubble is the average of the data points (*n* = 3).

Overall, the reservoir showed low taxonomic diversity, dominated by thermophilic and anaerobic taxa. The southern section of the Luno segment, represented by wells A13, A17, A18, and A19, showed lower microbial diversity compared to the rest of the reservoir (Figure 2A). The dominant taxa across all reservoir samples belonged to the genera *Thermoanaerobacter*, *Thermococcus*, and *Halomonas* (Figure 2B), which together accounted for the majority of 16S rRNA gene sequences.

**FIGURE 3:**
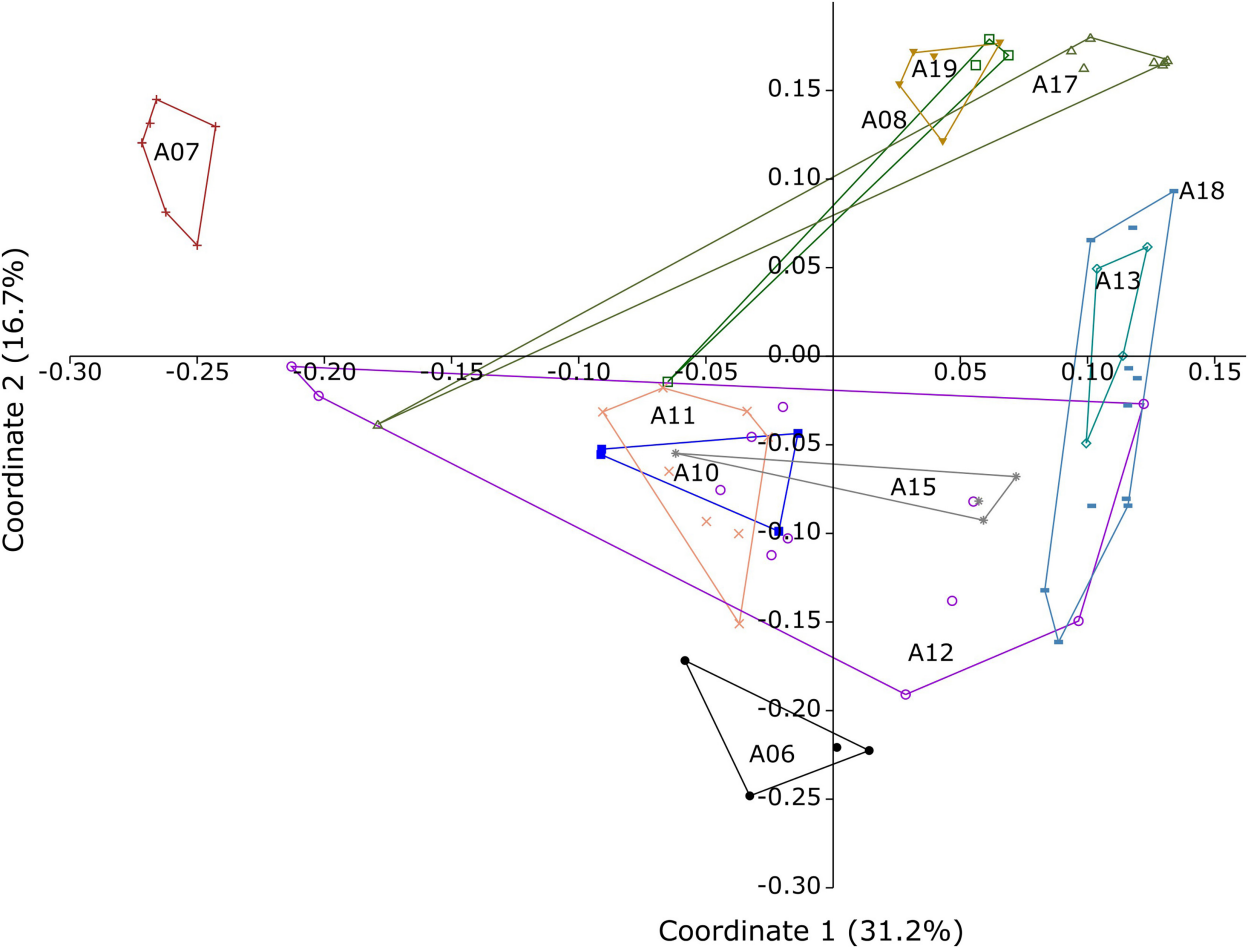
Principal coordinates analysis (PCoA) plot of microbial communities obtained from the produced fluids from Edvard Grieg reservoir, based on Bray-Curtis dissimilarity. Different symbols and colors denote the wellheads from which samples were collected. Axis 1 explains 31.2% of the variance, and Axis 2 explains 16.7%. The microbial community clustering pattern reflects the geographic and geological features of the reservoir, shown in Figure 1.

The reservoir exhibits local variation in microbial community composition (Figure 3). In the PCoA plot (Coordinates 1 and 2 explaining 31.2 and 16.7% of the total variance, respectively), samples from well A07, located in the northernmost part of the reservoir within the Tellus segment, cluster distinctly from the other wells, indicating pronounced compositional differences. These patterns are supported by PERMANOVA analysis based on Bray-Curtis dissimilarities, which revealed significant differences among wells (*p* < 0.05) (Supplementary Table S1). The distinct clustering of well A07 suggests that its microbial community differs substantially from the remainder of the reservoir, potentially reflecting its geographical isolation or differences in the geological characteristics of the Tellus segment. Geological and geochemical reservoir parameters (e.g., porosity, permeability) were not available, so these factors cannot be quantitatively correlated with the microbial differences.

Because bacterial and archaeal communities are able to adapt to the extreme conditions in oil reservoirs and potentially affect biogeochemical processes such as sulfur and methane cycling, we constructed a phylogenetic tree analysis of 16S rRNA genes (Supplementary Figure S1), focusing on extremophiles, sulfate reducers, and methanogens. For Bacteria, sulfate-reducing groups like Desulfobulbales, Desulfobacterota, Desulforomonadales, and Desulfovibrionales are present alongside extremophilic lineages such as Thermotogota and Thermonaerobacteraceae. Methanogens and other extremophiles are abundant among Archaea, represented by groups like Methanobacteriota and Thermococci.

We examined the relationship between microbial composition and water cut. The generalized additive model (GAMs) identified 38 ASVs significantly associated with water cut (*p* < 0.05) (Supplementary Table S3). Many of these ASVs belong to genera previously highlighted in the reservoir, including the thermophiles *Thermoanaerobacter*, *Thermococcus*, *Caminicella*, and *Petrotoga*, and halophiles such as *Halomonas*. PERMANOVA and the Mantel test showed a trend (*p* = 0.06) in associating water cut with microbial community but did not reach statistical significance (Supplementary Table S3). NMDS ordination (Supplementary Figure S2) revealed a clear clustering of ASVs based on water cut, with a stress value of 0.117, indicating a good fit of the microbial composition to the water cut data.

### 3.2 Impact of water breakthrough

**FIGURE 4:**
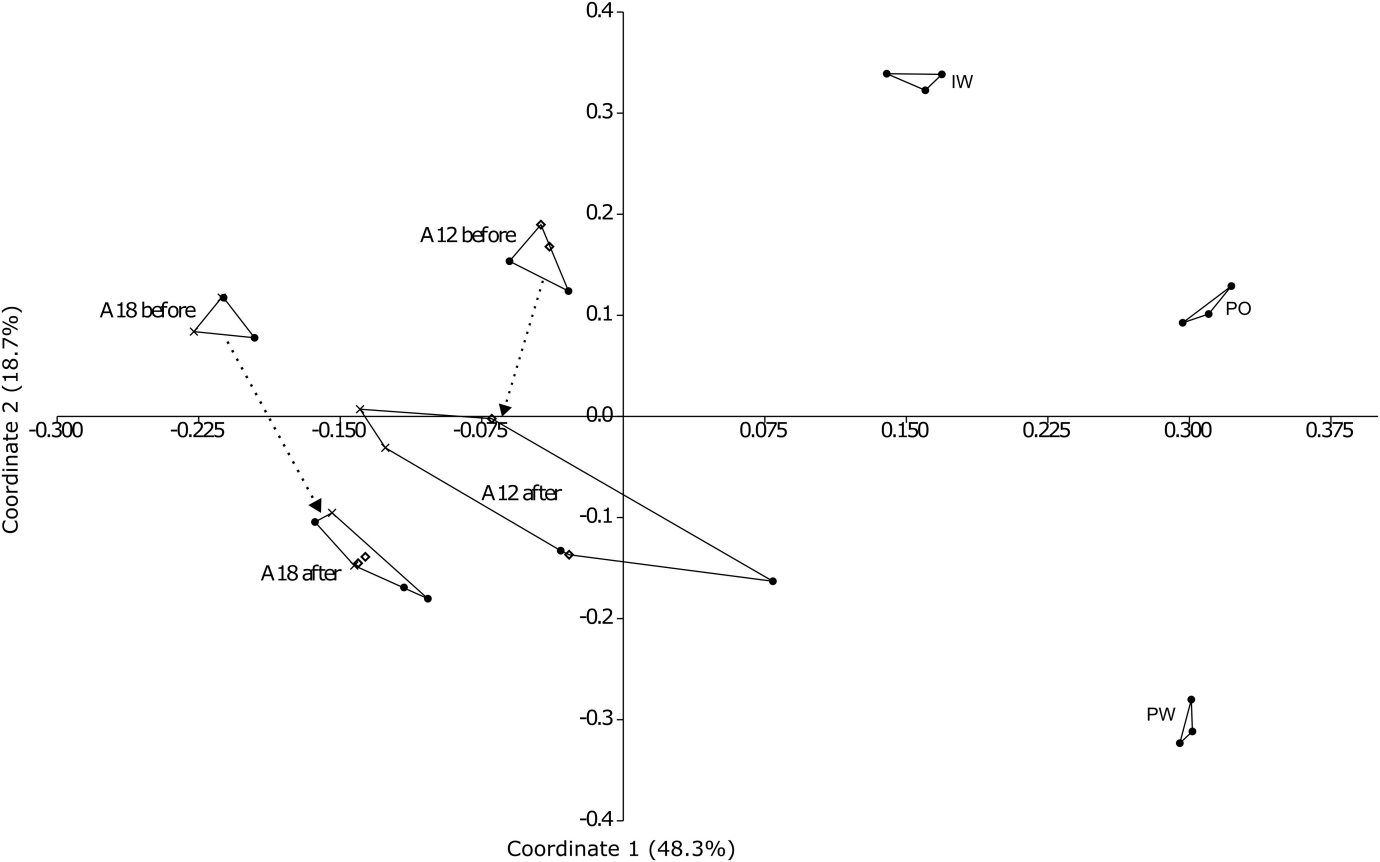
The effect of the water breakthrough on the microbial community. PCoA plot with Bray-Curtis similarity index of the two reference wells production fluids (PF) before and after water breakthrough, injection water (IW), production water (PW), and produced oil (PO). The different symbols correspond to the biological duplicates. Axis 1: 48.3%, Axis 2: 18.7% of explained variance.

Despite the limited sample size of only two wells experiencing water breakthrough, our results indicate that water breakthrough influences microbial community composition within the oil reservoir. The PCoA plot (Figure 4), with Coordinates 1 and 2 explaining 48.3 and 18.7% of the variance, shows significant differences in microbial community composition before and after water breakthrough (*p* < 0.05; Supplementary Table S4).

In PF samples from wells A12 and A18, PERMANOVA indicated statistically significant dissimilarities both before (*p* = 0.03) and after (*p* = 0.04) breakthrough. Although the numerical difference in *p*-values is small and the number of wells analyzed was limited to two, these results may suggest a potential homogenizing effect of water breakthrough, with microbial communities becoming slightly less distinct afterward. This interpretation is supported by the Bray-Curtis-based PCoA (Figure 4), which shows PF samples from wells A12 and A18 clustering more closely together after water breakthrough than before. Additionally, after water breakthrough, the microbial community structure in the PF sample from central well A18 (Figure 1) exhibits higher similarity to that from the PW (PERMANOVA - p 0.027 before vs. p 0.007 after breakthrough).

Notably, the IW, PO, and PW samples show very similar microbial community compositions and are statistically indistinguishable from each other (p 0.09, 0.10; Supplementary Table S4).

By contrast, the pre- and post-breakthrough PF originating from A12 and A18 wells display significant differences (p 0.004).

**FIGURE 5:**
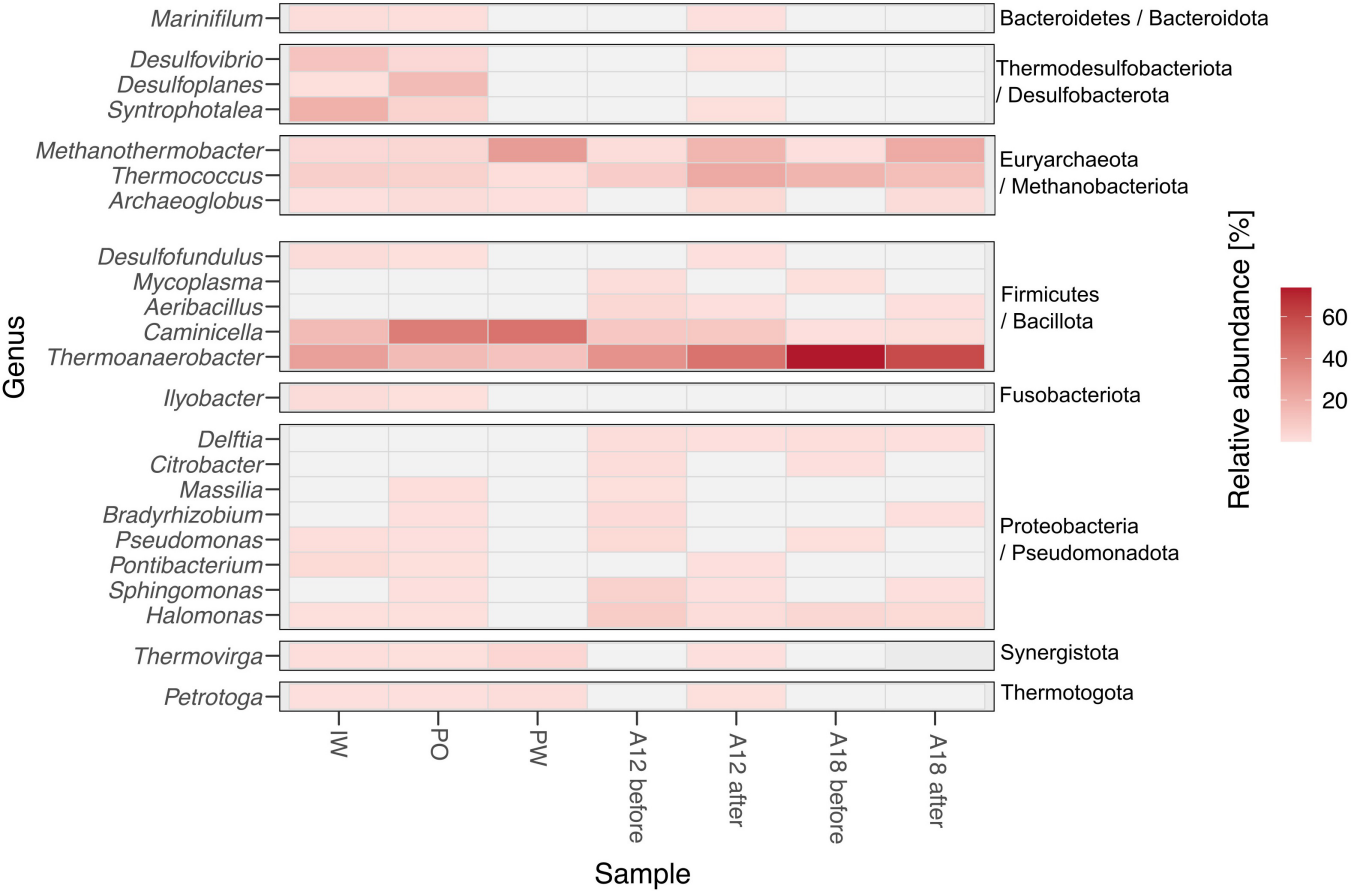
Matrix plot showing the relative abundance of 96% of the different genera present in the PF before water breakthrough, after breakthrough, in the injection water (IW), in the produced oil (PO), and in the production water (PW) samples (*n* = 3).

**FIGURE 6:**
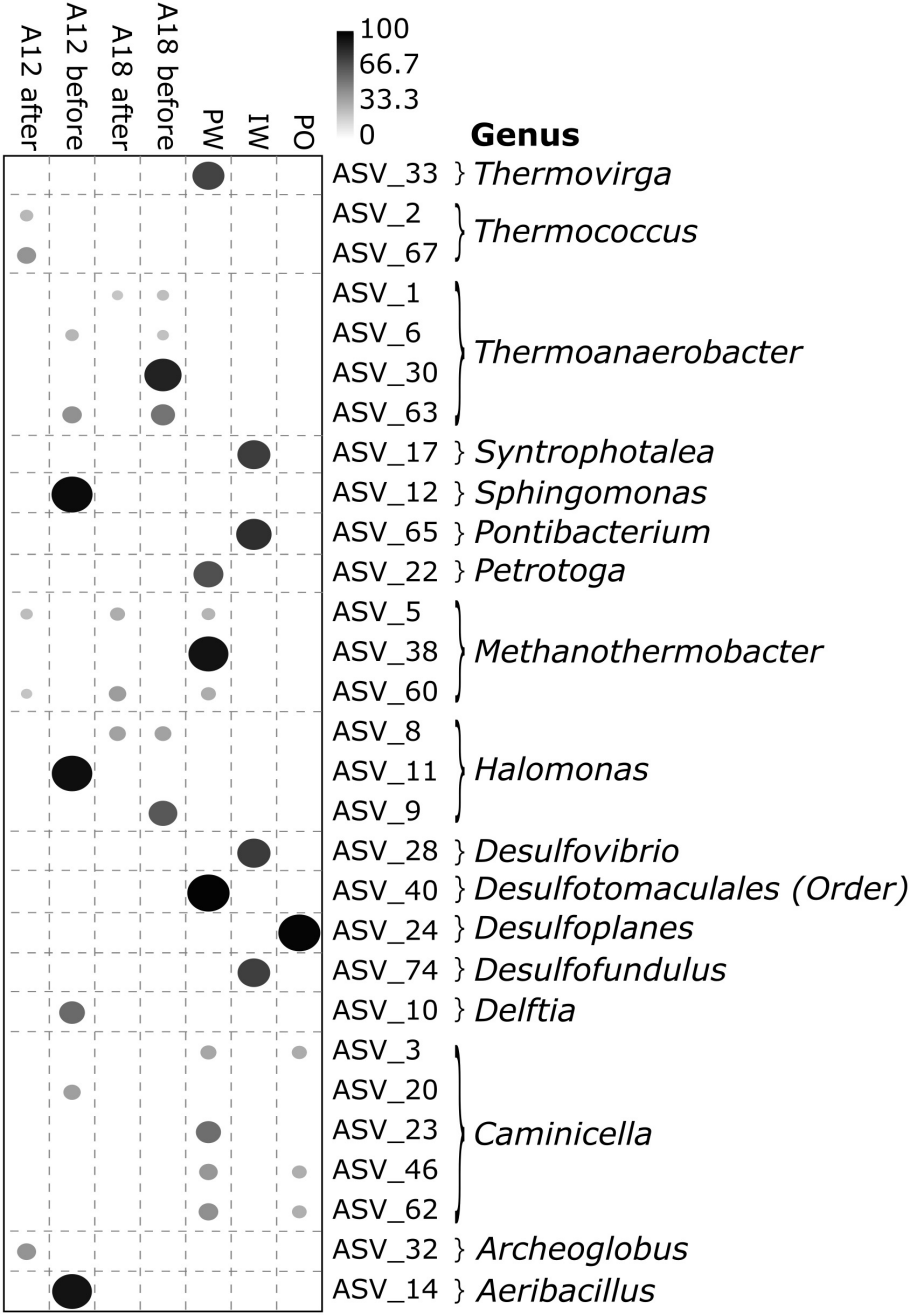
Indicator species analysis (IndVal) with a significance threshold of *p* < 0.05. The dataset was rarefied to 2,000 reads, and only taxa with a cumulative count of more than 100 reads were selected for analysis and considered particularly indicative of specific samples. Higher IndVal percentages indicate stronger associations and closer links of selected taxa to specific sample origins. A12 and A18 represent produced fluids before and after water breakthrough, respectively; IW denotes injection water, PO denotes the produced oil and PW denotes production water.

The detection of the four sulfate-reducing genera *Desulfofundulus*, *Archaeoglobus*, *Syntrophotalea*, and *Desulfovibrio* in the IW, as well as in PW and PF samples after water breakthrough, but not in PF samples from before breakthrough (Figure 5), suggests that these taxa are possibly introduced or likely their growth promoted by the injection water, although absence in earlier PF samples cannot be interpreted as absolute. This interpretation is supported by IndVal analysis (Figure 6), which revealed a significant association (*p* < 0.05) of IW samples with *Desulfofundulus*, *Desulfovibrio*, and *Syntrophotalea*. *Archaeoglobus* was significantly associated with PF from well A12 after breakthrough, consistent with its detection in post-injection samples. By contrast, *Thermoanaerobacter*, *Caminicella*, *Methanothermobacter*, and *Thermococcus* were consistently predominant across all samples. These taxa were nevertheless also present in the IW, suggesting that they may have been introduced into the IW by mixing with formation water and may persist or be recycled within the injection infrastructure, potentially surviving transient conditions. This highlights the complexity of distinguishing strictly indigenous taxa from circulating communities in managed reservoir systems.

**FIGURE 7:**
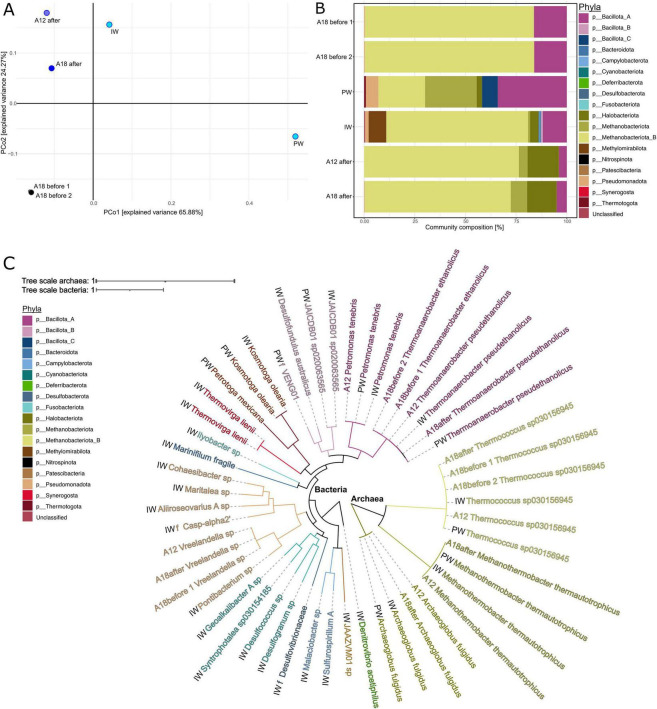
Metagenome and metagenome-assembled genome (MAG) characterization in terms of functional and taxonomic diversity and read abundances. **(A)** PCoA based on the abundance of 20,769 gene clusters. PF samples taken before water was injected into the reservoir are colored black, PF samples taken after water injection are colored dark blue, and the samples of the water injection and water separator fluid are colored light blue. **(B)** Community composition of prokaryotic organisms in the six samples based on coverage of extended rpS3 gene sequences. The proportion of phyla is shown by different colors. **(C)** Phylogenetic tree of metagenomic assembled genomes (MAGs). Nodes are colored according to the phyla of the MAG. Acronyms indicate sample origin: injection water (IW), production water (PW), and PF from wells A12 and A18.

Metagenomic analysis revealed a microbial community composition largely consistent with the 16S rRNA data. Community structure varied across samples, with distinctly different compositions observed before and after water breakthrough, as well as between the PW and IW samples (Figure 7). Across all samples, 248,889 genes were predicted, clustering into 177,477 species-level groups (Coelho et al., 2022), of which 20,769 were present in more than one sample. Notably, 28% of these shared clusters were detected in all samples (Supplementary Figure S3).

Following the water breakthrough, PF samples showed increased compositional variability and clustered closer to the IW sample, suggesting a shift in microbial community structure. However, interpretation of within-group dissimilarity is limited by the fact that pre-breakthrough samples originate from a single well, whereas post-breakthrough samples include two different wells (A12 and A18). The PW sample formed a distinct cluster, emphasizing its unique microbial composition. Although the dataset is statistically underpowered, these observations suggest that water breakthrough influences microbial community structure but does not completely overprint the reservoir’s putative microbial community.

MAG quality assessment (Supplementary Table S6 and Supplementary Figure S4) showed that 27 out of 51 metagenome-assembled genomes (MAGs) met the high-quality threshold ( ≥ 95% completeness, < 5% contamination). The majority of high-quality MAGs were reconstructed from the IW sample (25), followed by the PW (10). Key taxa, including Methanobacter_B, Bacillota_A, and Pseudomonadota, were retrieved from all groups of samples. The relative abundance of MAGs (Figure 7B) indicates substantial variation in microbial community composition between different sampling points. Before water breakthrough, PF samples were dominated by *Thermococcus* (Methanobacteriota_B), with relative abundances reaching 69–83%. After water breakthrough, the proportion of *Thermococcus* decreased by 11%, reflecting a shift in community structure.

The genera *Archaeoglobus* (Halobacteriota) and *Methanothermobacter* (Methanobacteriota) were absent in the metagenomes of the PF samples before water breakthrough but appeared in the post-breakthrough PF samples. *Methanothermobacter*, however, was found in the amplicon data prior to breakthrough. This discrepancy likely reflects differences in sequencing depth and detection limits between the two approaches, particularly under low-biomass conditions. The presence of *Methanothermobacter* in the amplicon data suggests that this taxon was already present at low abundance prior to water breakthrough but was not recovered in the corresponding metagenomes. Given its temperature optimum of 55–65°C and strictly anaerobic lifestyle (Boone, 2015), we assume that *Methanothermobacter* likely originates from the reservoir despite being associated with the IW. Taxa from the phyla Pseudomonadota and Bacillota_A were present across all samples but contributed differently to the overall community composition. Notably, the relative abundance of Bacillota_A, particularly RUG420 (Supplementary Figure S6), declined 3.6-fold after water breakthrough, indicating that this taxon may have been negatively affected or outcompeted as a result of the environmental changes caused by the injection process.

Phylogenetic analysis (Figure 7C) illustrates the taxonomic diversity of the microbial communities, including representatives from Euryarchaeota (Methanobacteriota), Desulfobacterota, Firmicutes (Bacillota), and Thermotogota. The detection of Desulfobacterota in both IW and PW supports the hypothesis that sulfate-reducing communities are introduced or stimulated by seawater injection, as seawater (a major component of IW) provides sulfate as an electron acceptor.

Metagenomic analysis revealed that PF from well A18 shared two viral strain clusters with PW and IW prior to water breakthrough, whereas 11 viral strains were shared after breakthrough, indicating a pronounced increase in viral overlap following water breakthrough (Supplementary Figure S5). All shared viral scaffolds were classified as *Caudoviricetes*, and three were tentatively assigned to the family *Autographiviridae* with low confidence. Viruses belonging to these taxa have previously been reported in deep subsurface environments, including trench sediments (Wang et al., 2025). The predicted hosts of the shared viral scaffolds were predominantly affiliated with thermophilic and anaerobic taxa characteristic of deep petroleum reservoirs, including members of the genera *Thermovirga*, *Thermoanaerobacter* and *Petrotoga* (Supplementary Table S11). Additional host assignments included thermophilic archaeal lineages not detected in our 16S rRNA and *rpS3* sequencing data, such as *Nitrosocaldus* and Woesearchaeales, as well as a limited number of taxa more commonly linked to surface or engineered environments (e.g., *Acinetobacter*).

### 3.3 Functional marker genes indicative of metabolic potential

**FIGURE 8:**
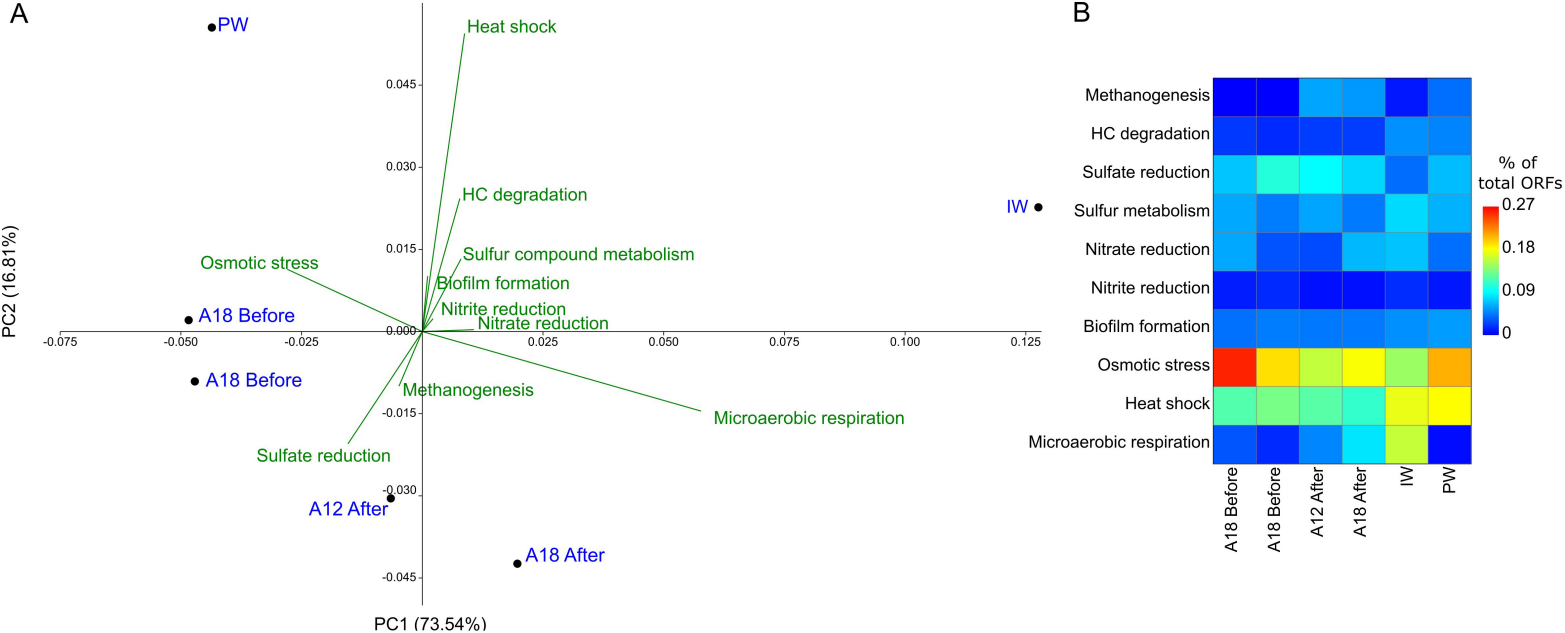
Distribution and abundance of functional genes relevant to oil reservoirs. **(A)** Principal component analysis (PCA) biplot of the ORFs of interest, with green lines indicating the direction of samples where the functional genes were found. **(B)** Heatmap showing the percentage of gene hits for the ORFs of interest relative to the total number of ORFs in each sample. A12 and A18 represent produced fluids before and after water breakthrough, respectively; IW denotes injection water, and PW denotes production water.

The ORFs selected from the metagenomes (Supplementary Table S2) revealed distinct patterns in functional gene abundance across samples taken before and after water breakthrough, highlighting shifts in microbial metabolic potential and stress responses associated with environmental changes (Figure 8). Methanogenesis-related ORFs were undetectable in samples collected before water breakthrough but were present in the PW and IW and post-water breakthrough PF. ORFs associated with hydrocarbon degradation were scarce and found mostly in post-water breakthrough PF samples.

Sulfate reduction-related ORFs were abundant in all samples but scarce in the IW sample. Nitrate and nitrite reduction were absent in the pre-breakthrough samples but present afterward, with a particular abundance of ORFs in the injection water itself. ORFs associated with osmotic stress were consistently detected across all samples, with the greatest abundance in one of the samples before the water breakthrough. Microaerophilic oxygen respiration-related ORFs were absent before water breakthrough and appeared in all subsequent samples, comprising 0.008% of total ORFs in PW, 0.045% in IW, and 0.034–0.054% in PF after breakthrough.

PERMANOVA results indicate that there is a statistically significant difference in ORF compositions between sample groups (p 0.0245), but the pairwise distance values show no significance based on ORFs composition (Supplementary Table S9).

## 4 Discussion

### 4.1 Regional differences and microbial composition

To the best of our knowledge, this study is the first to sample microbial communities directly from produced fluids, across multiple locations within the same hot oil reservoir with temperatures near the limit of biodegradation in hydrocarbon reservoirs (Wilhelms et al., 2001). The reservoir exhibited localized differences in microbial community composition, underscoring a crucial point: microbes could serve as indicators for understanding oil provenance and migration pathways within an oil reservoir, provided that temperatures remain within a biologically feasible range. This approach, as suggested by Zhang et al. (2020) and supported by our data, presents a promising, low-cost complement to traditional geological and geophysical methods, thereby enhancing our ability to monitor and understand reservoir dynamics in oil fields.

We assumed that, given the harsh DNA extraction protocol (Alibrandi et al., 2023), at least part of the DNA from endospores was also extracted and thereby sequenced with the total environmental DNA. Extreme reservoir conditions result in low microbial abundance of 10^2^–10^3^ cells × mL^–1^ and, consequently, low DNA yields (Alibrandi et al., 2023). Direct cell counting is impractical due to the high affinity of crude oil for fluorescent dyes, resulting in background signals from the oil being stronger than those from the microbial cells, thereby hampering reliable detection (Kallmeyer, 2011; Lloyd et al., 2013).

Microbial diversity across all wells of the reservoir was generally low. The oil samples from the southern segment (A13, A17, A18, A19) exhibit an even more pronounced reduced diversity. While the cause of this pattern remains unclear, the lack of correlation between water cut data and diversity indices suggests that local lithology, rather than water content, may be responsible. The microbial taxa identified in our dataset align with previous findings from oil reservoirs. Genera commonly reported in earlier studies and also observed in our study include *Corynebacterium, Chryseobacterium, Sphingomonas, Pseudomonas, Thermoanaerobacter, Methanothermobacter, Thermococcus, Petrotoga, Halomonas*, *Kosmotoga*, *Thermovirga*, *Archaeoglobus*, and *Caminicella* (Kaster et al., 2009; Wentzel et al., 2013; Head, 2017). Notably, despite being considered part of the indigenous community, *Thermovirga* was found exclusively in the samples after water breakthrough, in the PW, and in the IW. This might suggest that this taxon could have been introduced by the injection, or else, was present in the reservoir and found more suitable conditions in the water phase.

The predominant species in most samples was the strictly anaerobic bacterium *Thermoanaerobacter* and the archaeon *Thermococcus*. The MAGs’ taxonomic profiling based on the GTDB database (Figure 7C) attributed the genus *Thermoanaerobacter* to two different species, namely *Thermoanaerobacter ethanolicus* (Wiegel and Ljungdahl, 1981), a non-spore-forming bacterium in samples from well A18 before water breakthrough, and the spore-former *Thermoanaerobacter pseudoethanolicus* (Onyenwoke et al., 2007), in wells A12 and A18 after water breakthrough. However, FastANI analysis (Jain et al., 2018) of the *Thermoanaerobacter* genomes suggests that all strains belong to the same species, as their pairwise ANI values exceed 95%, the species threshold defined by Jain et al. (2018). The observed discrepancy in GTDB classification might stem from differences in genome completeness, with marker genes in the more complete *Thermoanaerobacter ethanolicus* genome influencing its placement. If the *Thermoanaerobacter pseudoethanolicus* genome from A18 were more complete, it might also be classified as *Thermoanaerobacter ethanolicus* (Supplementary Table S6 and Supplementary Figure S4). Alternatively, the missing genomic regions could reflect true species-level divergence. We considered endospore formation a potentially meaningful trait, as endospores are more resistant to extreme conditions such as high temperature and might therefore point to a deeper, possibly source rock-associated origin. However, given the high genomic similarity across strains, this distinction does not appear to reflect an actual separation at the species level. Consequently, the observed differences in sporulation capacity may be the result of annotation or genome completeness issues rather than ecologically or biogeographically meaningful divergence.

*Thermoanaerobacter* survives within a temperature range of 35–80°C, with an optimum growth temperature of 65–70°C (Rainey and Stackebrandt, 1993). Similarly, *Thermococcus* is an obligate anaerobic and hyperthermophilic archaeon, with a known growth temperature range of 60–105°C (Zhang et al., 2012). These temperature profiles are consistent with the reservoir’s thermal conditions, supporting the interpretation that these organisms are indigenous members of the subsurface microbial community rather than introduced via water breakthrough. Their presence across pre- and post-breakthrough samples further suggests that the high-temperature reservoir environment provides a stable ecological niche that sustains thermophilic populations despite operational perturbations.

### 4.2 Effects of water breakthrough on the community structure

PERMANOVA results indicated that the differences in community structure between IW, PO, and PW were not statistically significant, whereas pre- and post-breakthrough PF samples showed significant differences. Injection Water, Produced Oil, and Produced Water share common characteristics: they lack suspended particulates and are relatively homogeneous, providing no oil-water interfaces for microorganisms to colonize. In contrast, PF samples, which contain water, oil, and solids, exhibit significant detectable differences (*p* < 0.05; Supplementary Table S4) before and after water breakthrough, likely due to changes in fluid composition and microenvironments associated with the multiphase nature of the produced fluids. It should be noted that the temporal comparison is based on only two wells (A12 and A18), which limits the statistical power of these analyses. Consequently, observed trends, such as the potential homogenizing effect of water breakthrough, should be interpreted cautiously as indicative patterns within these wells rather than reservoir-wide effects. Despite this limitation, the observed trends are consistent across multiple complementary analyses (PCoA, PERMANOVA, and Bray-Curtis clustering), providing insight into microbial responses to water injection in the sampled regions.

The majority of taxa identified in the PF were also detected in the IW samples. However, four genera, *Syntrophotalea*, *Desulfovibrio*, *Desulfofundulus*, and *Archaeoglobus*, were exclusive to the IW and the PF post-breakthrough. All four genera are anaerobic, thermophilic or thermotolerant, and capable of sulfate reduction. Their presence in post-breakthrough PF can be attributed to their thermal resilience, given their high optimal growth temperatures (e.g., *Archaeoglobus* at 83°C; Klenk et al., 1997). Among them, *Syntrophotalea* plays a significant role in degrading organic compounds such as butanol and ethanol (Pereira et al., 2021). Additionally, *Syntrophotalea* can form syntrophic associations with methanogens, contributing to the degradation of crude oil, aromatic compounds, and alkanes (Gray et al., 2011).

We initially hypothesized that water breakthrough would significantly alter microbial distributions, leading to an overprinting of indigenous communities by injection-associated microbes. By contrast, reservoir engineers generally assume minimal mixing between injected water and oil, with water simply pushing oil ahead. However, while water breakthrough-associated microbial contamination was detected, extensive overprinting was absent. This is likely due to the short time since the water breakthrough (1 month) and the inherent challenges faced by allochthonous microbes in adapting to reservoir conditions.

Further supporting the limited extent of biological overprinting, our metagenomic data revealed viral genomic patterns that closely mirrored the 16S rRNA gene data. Specifically, viral communities in the PW and IW samples showed strong similarity and clustered together, while PF remained distinctly different (Supplementary Figure S5). Given the high mutation rates of viruses compared to cellular microorganisms, attributable to their smaller genomes and rapid replication cycles (Sanjuán and Domingo-Calap, 2016), it is highly unlikely that identical viral strains would independently emerge in physically and geochemically isolated environments.

Importantly, the similarity between PW and IW viral communities does not necessarily imply an exogenous origin of IW-associated viruses. Many inferred microbial hosts of the detected viral sequences are thermophilic taxa typical of the reservoir, including *Thermovirga*, *Thermoanaerobacter*, and *Petrotoga* (Supplementary Table S11), suggesting that a substantial fraction of the viruses detected in PW and IW are associated with reservoir-derived microorganisms circulating through the production and injection infrastructure. By contrast, the low viral signal observed in PF samples prior to water breakthrough is likely influenced by limited DNA recovery rather than a true absence of viral populations.

Microaerobic respiration genes detected in the data do not necessarily imply active oxygen respiration but rather that the associated microorganisms can tolerate oxygen. These genes were absent in pre-breakthrough PF but were present in post-breakthrough PF and IW samples (Supplementary Table S2). We argue that oxygen consumption in the reservoir is minimal, with injection water, despite being degassed, representing the primary source of oxygen. Alternatively, oxygen may also arise from internal subsurface processes, with abiotic reactions such as water radiolysis generating trace amounts of so-called “dark oxygen” in deep geological environments (Lin et al., 2005). Taken together, these findings support the interpretation that the taxa detected prior to water breakthrough reflect the indigenous microbial community of the oil reservoir.

### 4.3 Methodological applications

A major difference between the 16S rRNA results and the metagenome results is a major underrepresentation of the archaeal taxa in the 16S rRNA data set. The genus *Thermococcus* in Figure 2 seems less abundant than *Thermoanaerobacter*, whereas the metagenomic data in Figure 7B show the pattern reversed. This discrepancy highlights the known biases in 16S rRNA gene sequencing, where primer design often favors bacterial sequences, leading to poor amplification of archaeal 16S rRNA genes (Teske and Sørensen, 2008). By contrast, the *rps3* gene sequences obtained through the metagenomes provide a more accurate picture of microbial diversity. Our findings emphasize the need to complement 16S rRNA studies with metagenomic approaches if the study aims to quantitatively identify microbial community structure.

In a previous study on a cooler oil field in the North Sea, we observed that PF samples provide a more accurate reflection of reservoir conditions than PW (Alibrandi et al., 2025). In contrast, the present study reveals a greater similarity of the taxonomic composition between IW, PW, and PF (Figure 4). This resemblance may be explained by the higher reservoir temperature of Edvard Grieg compared to the North Sea field (80°C vs. 65°C) or by a larger proportion of formation water used in the injection process. Although IW includes seawater as well, taxa typically associated with seawater were scarce. We attribute this to the high-temperature treatment, which likely eliminates most seawater-derived organisms. Consequently, we assume that the IW primarily contains formation water microorganisms, which may explain the observed similarity in community composition across the different sample types.

Our findings contribute to the broader understanding of microbial ecology in high-temperature oil reservoirs and highlight the persistence of certain genera under extreme thermal conditions. While microbial research in petroleum systems has been ongoing for decades, its integration into routine oil field monitoring has remained limited, partly due to the past high DNA sequencing costs and partly because sequencing data were long regarded as scientifically interesting but not directly useful for operational monitoring or decision-making. This study reinforces the potential value of microbial data in reservoir characterization by demonstrating the distinct taxonomic profiles of indigenous versus injection-associated communities. Although our study captures only a short timeframe following water breakthrough, it emphasizes the importance of temporal resolution. Longitudinal studies tracking microbial dynamics over different operational stages, such as water flooding, breakthrough, and shut-in, could help clarify the long-term ecological consequences of water injections. By shedding light on microbial diversity and spatial structuring in a deeply buried, polyextreme environment, our results offer a foundation for more targeted investigations into microbial roles in reservoir processes and their potential relevance for management practices.

## Data Availability

The data presented in this study are publicly available. The data can be found here: https://www.ebi.ac.uk/ena/browser/, accession number PRJEB81118.
