# Supplementary material for: Microbial diversity and community shifts in a petroleum reservoir under production: effects of water breakthrough and anthropogenic alterations
Source: Front Microbiol. 2026 Mar 19;17:1741638. doi: 10.3389/fmicb.2026.1741638 (PMC13044146; doi:10.3389/fmicb.2026.1741638)
Supplement: Supplementary file 1 [file Data_Sheet_1.docx]

***Supplementary material***

**Microbial Diversity and Community Shifts in a Petroleum Reservoir under Production: Effects of Water Breakthrough and Anthropogenic Alterations**

Armando Alibrandi^1,2^, Julia Plewka^2^, Rolando di Primio^3^, Alexander Bartholomäus^1^, Aurèle Vuillemin^1^, Alexander J. Probst^2^, Jens Kallmeyer^1^

(1) armando.alibrandi@gfz-potsdam.de, GFZ German Research Centre for Geoscience, Section Geomicrobiology, Germany.

(3) University of Duisburg-Essen, Group of Environmental Metagenomics, Germany

(4) Aker BP, Norway.

**Content:** supplementary methods, supplementary figures and tables, supplementary references.

**Supplementary figures**

- **Supplementary figure S1:** Phylogenetic analyses of 16S rRNA genes
- **Supplementary figure S2:** NMDS plot of the PF samples in relation to the water cut
- **Supplementary figure S3:** Intersection size and percent intersection ratio (proportion of shared cluster between the respective intersection) of the non-singleton gene cluster (total 20,769) between the six metagenomes
- **Supplementary figure S4:** Completeness and contamination of metagenome assembled genomes (MAGs)
- **Supplementary figure S5:** Edvard Grieg viral strain clustering
- **Supplementary figure S6:** Normalized coverage of extended rpS3 gene sequences

*
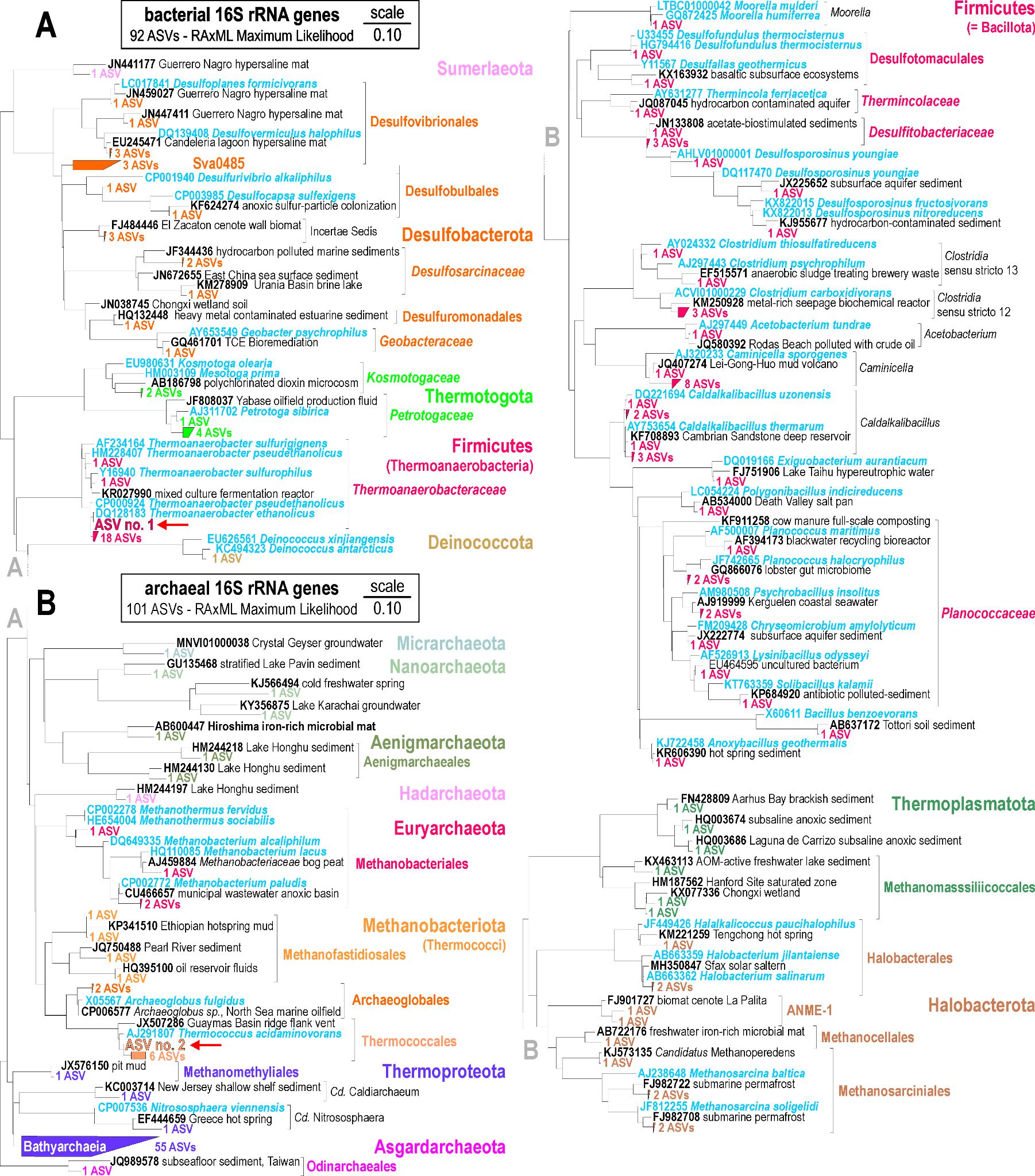
***Figure S1: Phylogenetic analyses of 16S rRNA genes**. RaxML Maximum Likelihood phylogenetic tree of partial 16S rRNA genes (V4 hypervariable region) taxonomically assigned to (**A**) sulfate reducers and extremophiles among Bacteria and (**B**) methanogens and extremophiles among Archaea. Taxonomic assignments focused on groups relevant to oil reservoir environments, specifically targeting sulfate-reducing bacteria and extremophilic lineages within Bacteria, and methanogens and extremophilic groups within Archaea.

**
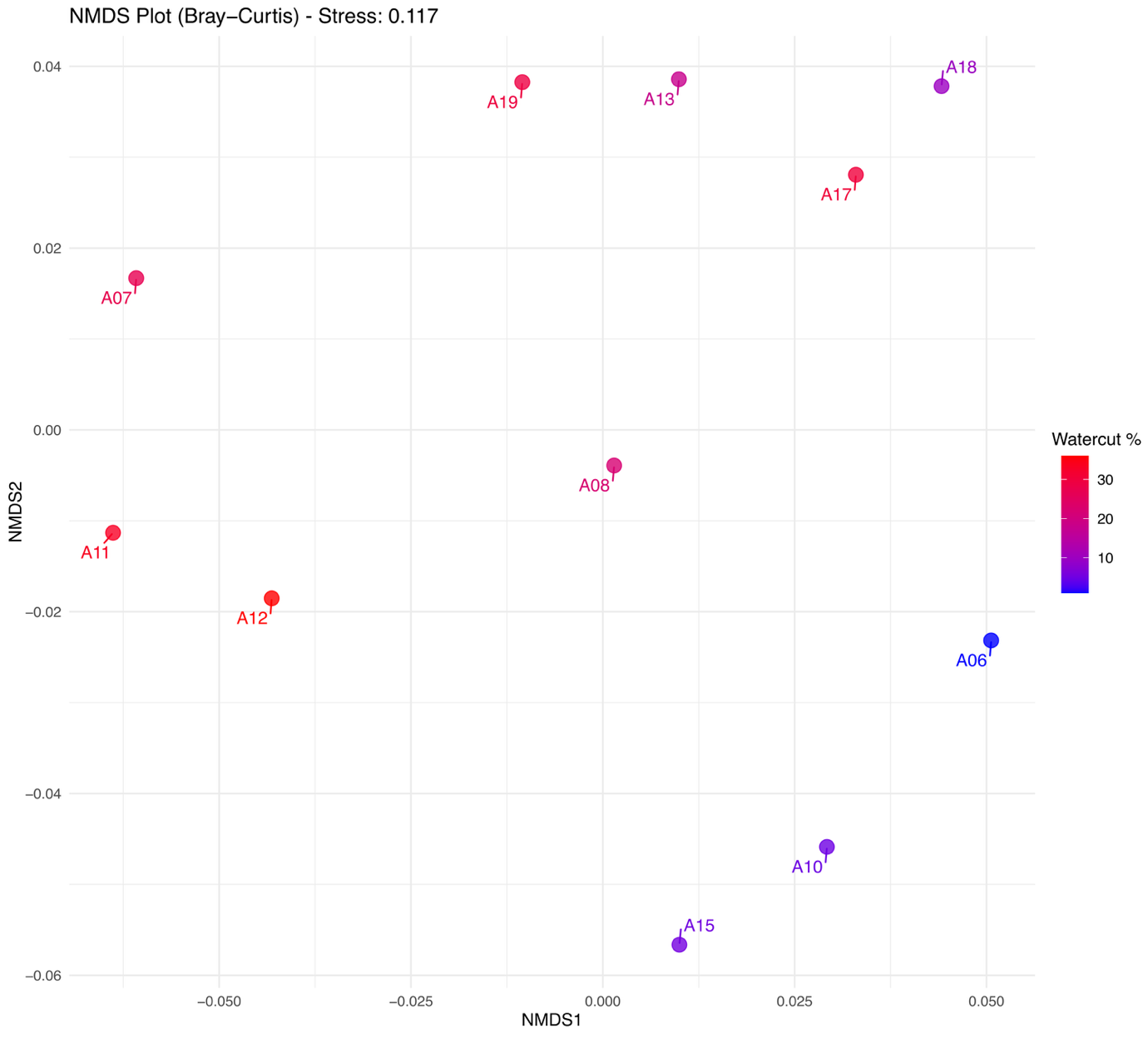
Supplementary Figure S2: Non-metric multidimensional scaling (NMDS) plot based on Bray-Curtis dissimilarity, illustrating microbial community composition across samples in relation to the water cut.** Each point represents a sample, colored according to its Water cut value (blue = lower Water cut, red = higher Water cut). The proximity of points indicates the similarity of microbial communities, with closer points showing more similar compositions. The stress value (0.117) reflects the goodness of fit for the ordination. A gradient in color distribution suggests a potential relationship between Water cut and microbial community structure.

**
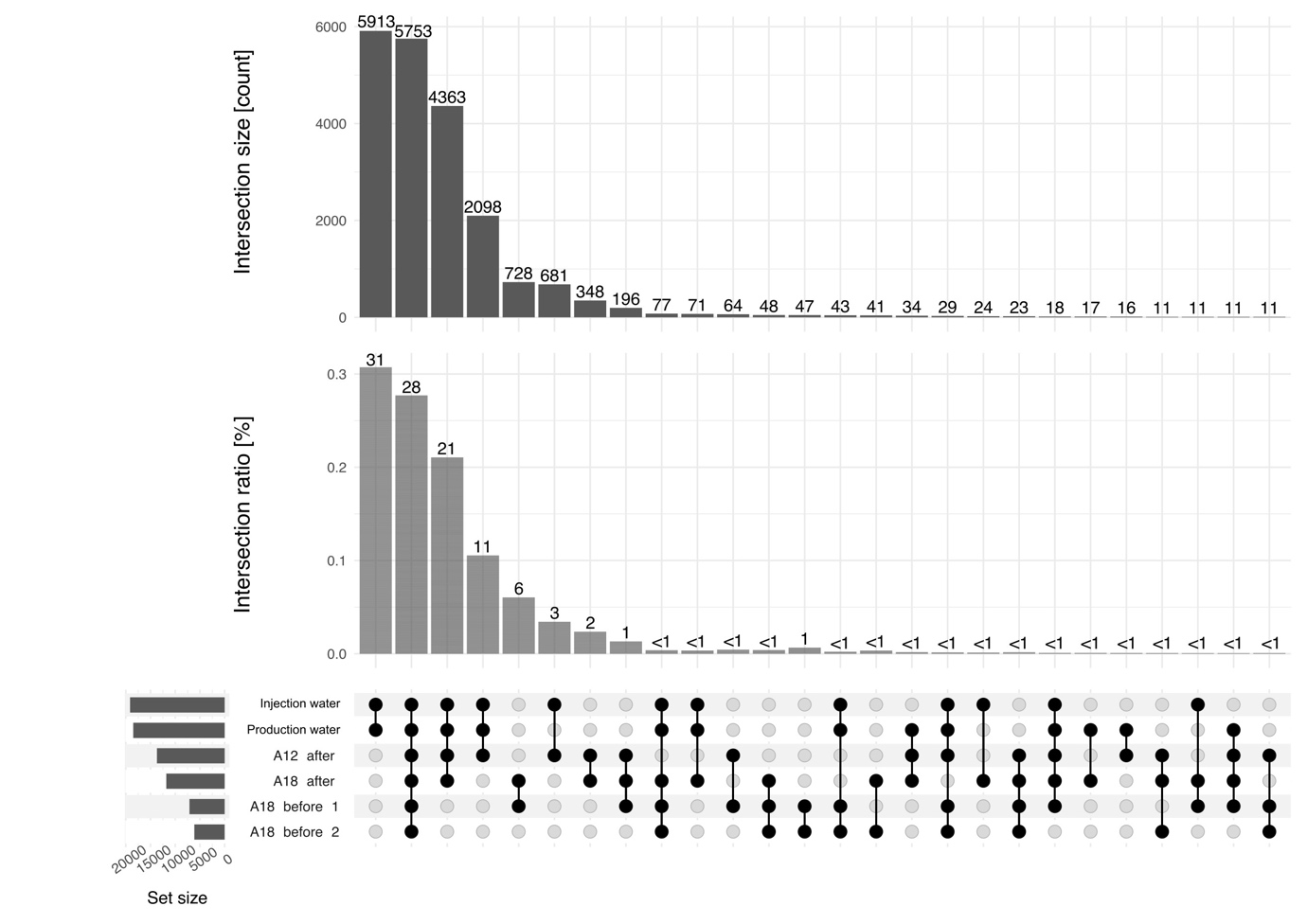
Supplementary Figure S3: Intersection size and percent intersection ratio (proportion of shared cluster between the respective intersection) of the non-singleton gene cluster (total 20,769) between the six metagenomes.** All sets with more than 10 shared clusters are shown.

 **Supplementary Figure S4: Completeness and contamination of metagenome assembled genomes (MAGs).** Red rectangular marks thresholds for high-quality MAGs. Color indicates phyla of MAG.


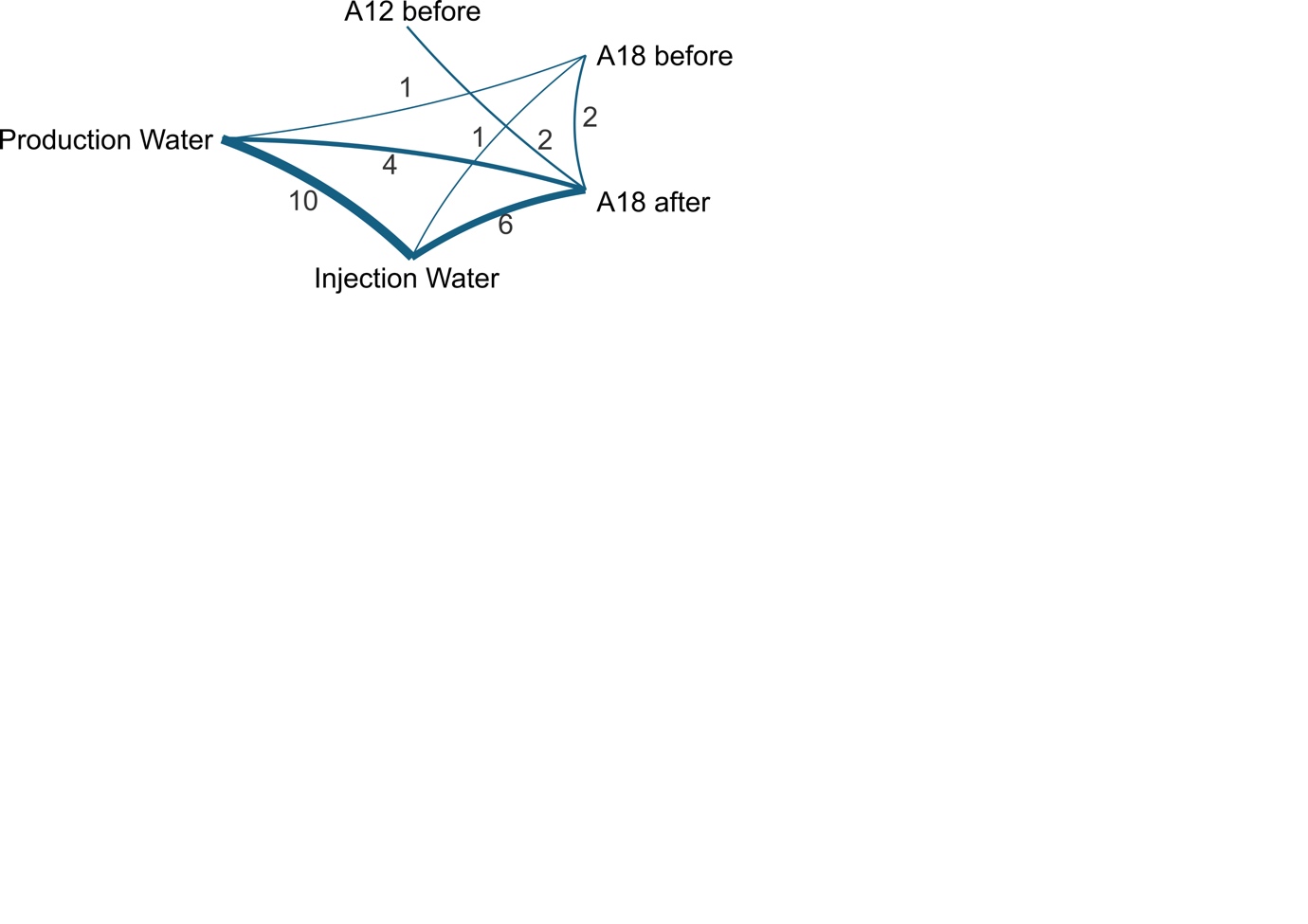


**Figure S5: Edvard Grieg viral strain clustering.** Line thickness corresponds to shared viral strain clusters (also displayed as numbers on the lines).

**
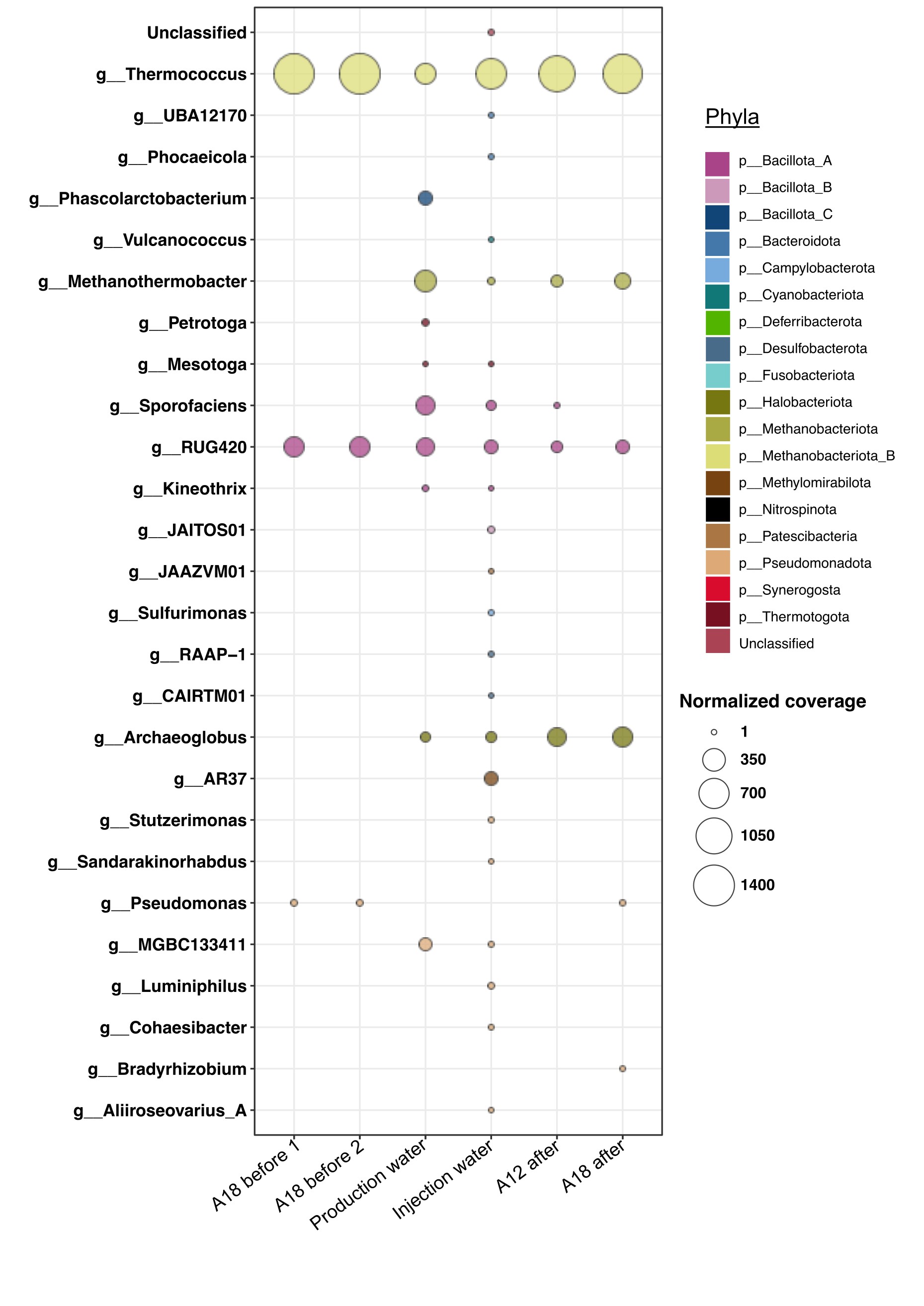
Supplementary figure S6: Normalized coverage of extended rpS3 gene sequences summarized for the assigned genera.** Bubbles are coloured according to phyla.
